# Zoledronic acid in the management of mesothelioma - a feasibility study (Zol-A Trial): study protocol for a randomised controlled trial

**DOI:** 10.1186/s13063-018-2851-9

**Published:** 2018-08-29

**Authors:** Duneesha de Fonseka, Anna Morley, Louise Stadon, Emma Keenan, Steven Walker, Sarah Smith, John E. Harvey, R. Ashley Cox, Adam Dangoor, Charles Comins, Christine Rogers, Anthony Edey, Alfredo Addeo, Nick A. Maskell

**Affiliations:** 1 Academic Respiratory Unit, University of Bristol, Bristol, UK; 2 Respiratory Research Unit, North Bristol NHS Trust, Bristol, UK; 3 North Bristol NHS Trust, Bristol, UK; 4 Royal United Hospitals Bath NHS Foundation Trust, Bath, UK; 5 Bristol Haematology and Oncology Centre, Bristol Royal Infirmary, Bristol, UK; 6 Clinical Trials and Evaluation Unit, Bristol, UK; 7 Radiology Department, North Bristol NHS Trust, Bristol, UK

**Keywords:** Mesothelioma, Malignant pleural mesothelioma, Zoledronic acid, Mesothelin, PET-CT

## Abstract

**Background:**

Nitrogen containing bisphosphonates such as zoledronic acid (ZA) are known to contain certain anti-cancer properties. These have been investigated in the past in various cancers such as breast, prostate and colon. ZA in particular has shown promising results in pre-clinical studies. We propose a multicentre double-blind randomised controlled feasibility study to assess the recruitment and acceptability of ZA/placebo alongside chemotherapy in malignant pleural mesothelioma (MPM).

**Methods:**

Patients will be recruited for a 13-month period from October 2016 to November 2017. Eligible patients will be identified via the regional mesothelioma multidisciplinary team meeting. Those who receive chemotherapy will be randomised to receive either ZA or placebo alongside their chemotherapy. Those who decline chemotherapy will be offered to join the trial on the non-randomised open-labelled arm of the trial. Patients will receive a maximum of six cycles of ZA/placebo, at three-weekly cycles. All patients will be followed up for six months from randomisation.

Semi-structured interviews to gather data on acceptability of trial procedures, tolerability of ZA and other relevant information will take place after the participants have completed their six cycles of treatment. For a better understanding about non-participation in mesothelioma trials we also aim to interview those who decline to take part in the trial.

**Discussion:**

The qualitative and quantitative data gathered in this feasibility trial will hopefully pave the way to designing a robust full phase III trial to investigate the potential synergistic effect of ZA and current standard treatment for MPM, cisplatin-pemetrexed combination chemotherapy.

**Trial registration:**

ISRCTN Registry, ISRCTN45536692. Registered on 9 August 2016. EudraCT no. 2015–004433-26.

**Electronic supplementary material:**

The online version of this article (10.1186/s13063-018-2851-9) contains supplementary material, which is available to authorized users.

## Background

Malignant pleural mesothelioma (MPM) is an aggressive and fatal tumour of the pleura that usually develops as a consequence of previous asbestos exposure. Health and Safety Executive data for 2012 show that mesothelioma caused 2535 deaths in the UK (http://www.hse.gov.uk/Statistics/causdis/mesothelioma/mesothelioma.pdf). Median survival without treatment for MPM is approximately 12 months; even with treatment the five-year survival rate still remains low at 5% [1]. The currently accepted standard treatment of cisplatin-pemetrexed combination chemotherapy only provides a modest survival benefit of three months when compared to single agent cisplatin alone, with only 40% of patients receiving chemotherapy responding to the treatment [2]. Hence there is a clear need to find novel treatments that work in MPM. As with many other cancers, the limelight is currently on immunotherapy and check-point blocking agents. Several Phase I to III trials are currently investigating the effect of these novel therapies in MPM (NCT03063450, NCT02588131, NCT02959463). To date, the best evidence with a good survival benefit is shown with bevacizumab, an anti-angiogenic targeting vascular endothelial growth factor [3]. This treatment is not without its side effects and to date has not been adopted by the National Institute for Health and Care Excellence in the UK.

Bisphosphonates are a synthetic analogue of naturally occurring pyrophosphate. Bisphosphonates are commonly used in the treatment of osteoporosis and other bone disorders, such as Paget’s disease, due to their action on inhibiting osteoclast mediated bone resorption [4]. Nitrogen containing bisphosphonates (n-bisphosphonates) have been shown to inhibit various epithelial cancer cells in vitro, by inhibiting the mevalonate pathway [5]. Potential anti-tumour activity of bisphosphonates includes reduced tumour angiogenesis, reduced tumour cell proliferation, migration, invasion and adhesion, increased tumour cell apoptosis and increased cytotoxicity of gamma-delta T cells, which subsequently leads to reduced tumour vascularisation [6].

Several studies using n-bisphosphonates, particularly zoledronic acid (ZA), have shown a survival benefit in patients with breast cancer [7, 8]. In vivo studies on mice inoculated with mesothelioma cells and treated with bisphosphonates have shown a significant survival advantage [9], supporting the direct anti-cancer properties of bisphosphonates in mesothelioma. Similar results have been seen in other in vivo studies of murine models inoculated with small-cell and non-small-cell lung cancer, both showing a reduction in tumour burden and increased survival in mice treated with n-bisphosphonates [10, 11].

ZA is known to be a potent nitrogen-containing bisphosphonate which has bone-independent anti-tumour activity. In addition, when combined with certain chemotherapy agents such as paclitaxel, etoposide, cisplatin and irinotecan in lung cancers, it has an even greater synergistic effect in induction of apoptosis in vitro [10].

As human studies investigating the synergistic effect between ZA and chemotherapy do not exist, the optimum timing of ZA in relation to chemotherapy is still unknown. Murine models using subcutaneously injected breast cancer cells have shown the greatest effect on increasing apoptosis; reducing proliferation and neovascularisation was seen when the cytotoxic drug was given 24 h after ZA [6].

A study by Jamil et al. [12] recently investigated the role of single agent ZA in a small cohort of patients with MPM who have either completed chemotherapy or were too frail to receive chemotherapy. They demonstrated some benefit with ZA, where there was a 37.5% rate of clinical benefit (progression-free survival and stable disease). Another study at our centre by Clive et al., looking at the role of ZA in malignant pleural effusions, demonstrated two patients with MPM who showed a reduction in tumour bulk on radiology [13] after receiving two doses of ZA intravenously.

A double-blind multicentre randomised controlled trial (RCT) would be best placed to investigate the hypothesis that treatment with the n-bisphosphonate ZA, in addition to the standard chemotherapy (pemetrexed and cisplatin), confers a survival benefit to patients with MPM compared to chemotherapy alone. We propose a feasibility study before undertaking a full study to capture the data needed to inform a definitive phase III trial. In the Zol-A trial, we are aiming to randomise 50 patients to receive either ZA or placebo alongside chemotherapy. In our feasibility study, a non-randomised third group will consist of patients who are fit for chemotherapy but have declined chemotherapy. These patients would be offered ZA in isolation. Semi-structured interviews as a part of the trial will help us to understand patient experiences, as well reasons behind patients’ decisions to decline chemotherapy or participating in the trial.

## Methods

### Feasibility outcomes

Our primary feasibility outcome is randomising 50 patients over a 12-month period. In addition, we have a number of secondary feasibility outcomes largely exploring the acceptability of recruitment procedures, consent and randomisation, data collection methods, acceptability of ZA in MPM patients, and the optimal timing and location for ZA administration. Qualitative analyses (QA) using semi-structured interviews are planned for patients who consent to the trial (in the randomised and non-randomised arms) and those who decline to participate in the trial, who agree to participate in the interviews. Other feasibility outcomes include: quantification of drop-out and data completeness rates; estimates of outcome event rates, e.g. survival times; measures of mean response and outcome variance (continuous variables such as quality of life) and confidence intervals around estimates of proportions; and categorical variables such as recruitment rates to use for calculating full trial size and number of sites for a phase III trial.

### Study overview

The trial is funded by the National Institute for Health Research (NIHR), Research for Patient Benefit (RfPB) funding stream. The trial protocol and related documents were reviewed by the Cambridge East Research Ethics Committee (REC) and the necessary approvals were granted in May 2016 (Reference no. 16/EE/0105). The trial is registered with ISRCTN, trial registration number 45536692. The trial is sponsored by North Bristol NHS Trust.

### Study design

The Zol-A trial is a multicentre, double-blind RCT assessing the feasibility of randomising 50 patients over a 12-month period across three NHS sites in the South West region of the UK. The lead centre is North Bristol NHS Trust (NBT) while Bristol Haematology and Oncology Centres at University Hospitals Bristol NHS Foundation Trust and Royal United Hospitals NHS Foundation Trust in Bath are the other two recruiting centres.

### Participant identification

Potential participants will be identified by the principal investigators (PI) across the three sites, primarily from the local lung cancer/mesothelioma multidisciplinary team (MDT) meetings. The regional mesothelioma MDT meeting is held at the lead centre NBT, which is led by the chief investigator for the study. All new cases of MPM from across the region (including the three hospitals taking part in the trial) are discussed at this meeting. Patients who meet the eligibility criteria will be identified as potential participants.

### Pre-screening, screening and recruitment

All patients with a new diagnosis of mesothelioma will be pre-screened for the trial. Potential participants who are eligible will be invited to take part in the trial. Patients will be given the patient information leaflet (PIL) at the time of their diagnosis. Patients will be next approached at the time of their first oncology clinic visit for re-discussion of the trial; those who are happy to take part will be invited to consent at this point. Doctor consent will be obtained by those clinicians enrolled on the delegation log.

### Eligibility criteria

#### Inclusion criteria

If the participants meet all the following criteria they will be eligible for the study:

- Histocytologically confirmed diagnosis of MPM
- World Health Organization (WHO) performance status (PS) 0–1
- Eligible for first-line chemotherapy
- Ability to give informed consent

#### Exclusion criteria

If the participants meet any of the criteria below, they will not be eligible for the study:

- Not fit for chemotherapy due to PS or other co-morbidities
- Previous chemotherapy for MPM
- Intravenous bisphosphonate therapy in the preceding three months
- Significant renal disease defined as an eGFR < 30 mL/min within the preceding four weeks
- Current hypocalcaemia receiving treatment or evidence of hypocalcaemia within the preceding six weeks
- Age < 18 years
- Severe untreated dental caries
- Concomitant participation in another drug trial for MPM
- Allergy to 18-fluorodeoxyglucose used for PET scans
- Women of child-bearing potential (defined as fertile, or following menarche and until becoming post-menopausal unless permanently sterile).

### Randomisation and blinding procedures

Patients will be allocated on a 1:1 basis to either the intervention (ZA) or placebo. The allocation will be blocked using varying block sizes and stratified according to histological subtype (epithelioid or cytological versus non-epithelioid) using web-based software provided by Sealed Envelope Ltd.

Participants and investigators will be blinded to the treatment received. The ZA or placebo will be provided in identically matched 100-mL 0.9% saline bags. The infusion bag will contain the participant trial ID and randomisation kit number. The allocation of treatment pertaining to the relevant randomisation kit number will remain within the pharmacies preparing the IMP/placebo.

### Randomisation code breaking and emergency unblinding

The code should only be broken in circumstances when knowledge of the IMP is required for treating the patient. The chief investigator has the primary right to break the blind if the circumstances warrant unblinding. In a non-emergency situation where unblinding is deemed necessary, the trial manager or the chief investigator will review the necessity of unblinding. In an emergency where unblinding is necessary, a 24-h rapid code-breaking service is available via the unblinding service provider based at Bristol Royal Infirmary (BRI) clinical trials pharmacy. The BRI pharmacy will hold the randomisation code list as well as restricted password-protected access to the web-based electronic randomisation services which would allow access to the randomisation codes for each participant.

### Trial interventions

#### Baseline assessment

A baseline assessment at the point of recruitment will capture demographic data, participants’ current medications and investigations undergone for the diagnosis of MPM. Participants will also have baseline bloods tests which include a full blood count, electrolyte levels (including adjusted calcium level, magnesium and phosphate), liver function tests and C-reactive protein (CRP) level. A research-specific blood test, serum mesothelin, will also be checked at the baseline assessment. Those receiving ZA/placebo alongside chemotherapy will then be randomised at the end of the baseline assessment. All participants will receive calcium supplementation from baseline assessment onwards, to prevent developing hypocalcaemia secondary to bisphosphonate therapy.

#### IMP/placebo schedule and administration

**Fig. 1:**
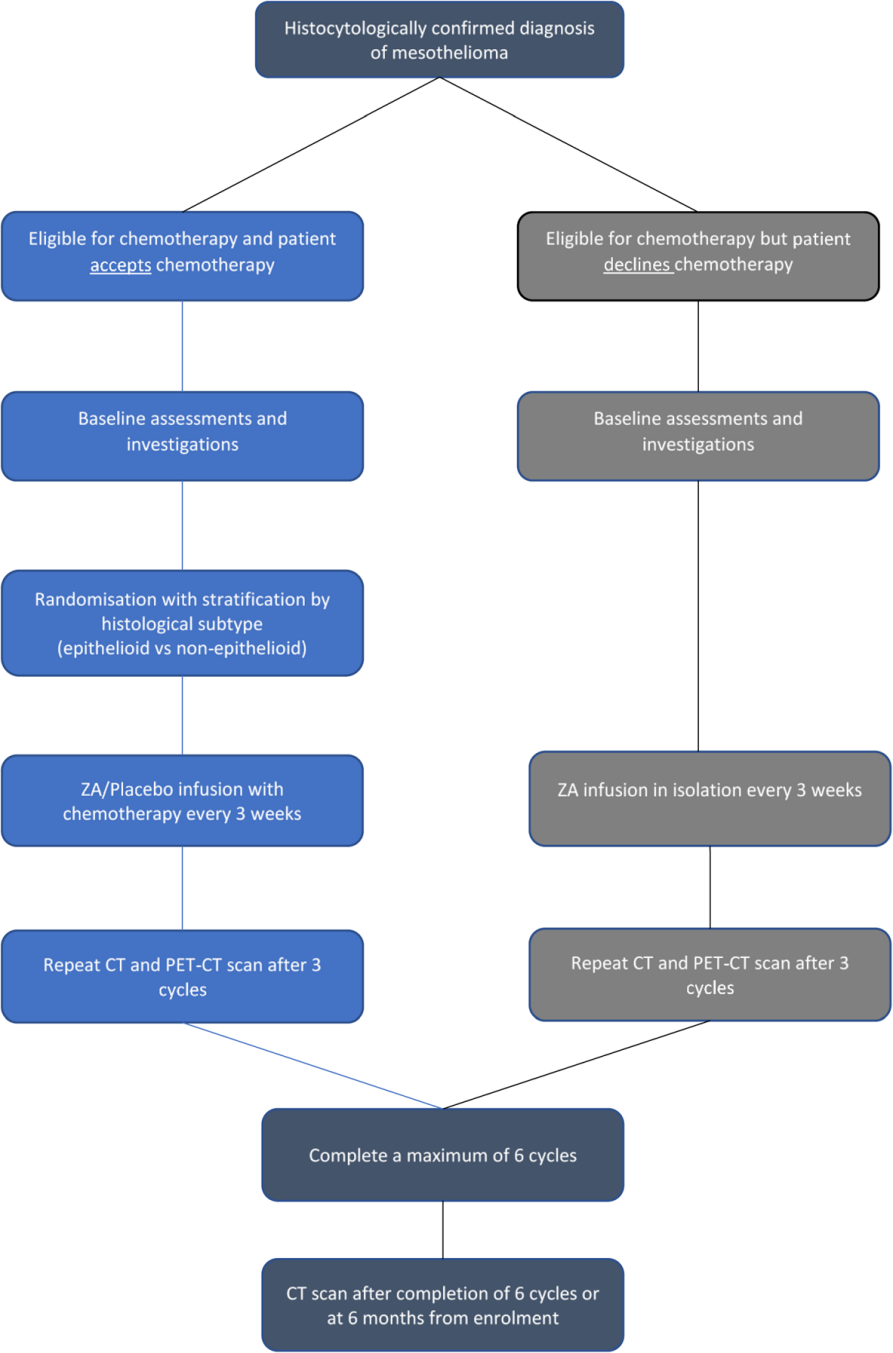
Trial *flow chart*

**Fig. 2:**
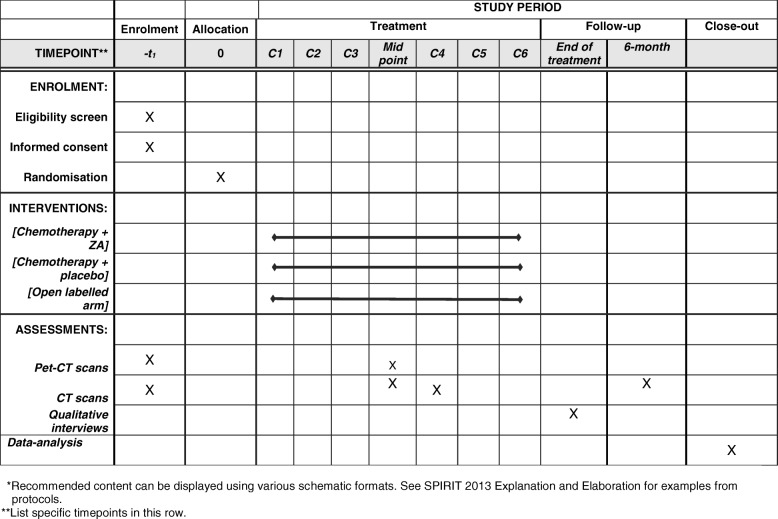
SPIRIT figure

Patients in both the randomised and non-randomised arms will receive up to a maximum of six cycles of ZA/placebo, at three-weekly intervals. Participants who stopped chemotherapy early, before the full six cycles, due to toxicity or other reason will stop IMP/placebo at the same time as their chemotherapy (Figs. 1 and 2).

ZA will be given in 100 mL of 0.9% saline over a 15-min period. The exact dose of the ZA will be dependent on their most recent renal function (see Table 1) which would be within the preceding seven days.

**Table 1:** Dose of ZA to be administered according to renal function

Renal function	Dose of ZA (mg)
eGFR ≥ 60 mL/min	4.0
eGFR 50–59 mL/min	3.5
eGFR 40–49 mL/min	3.3
eGFR 30–39 mL/min	3.0
eGFR < 30 mL/min	0.0

#### Follow-up assessments

All participants will have a follow-up appointment before their next cycle of chemotherapy (See Additional file 1). For patients in the randomised arm this often corresponds to their pre-chemotherapy oncology assessment visits. Data regarding adverse effects and other symptoms will be captured at the follow-up appointment. If the blood tests identify any electrolyte disturbances, additional supplementation will be prescribed as required.

A final follow-up appointment will take place at six months from enrolment (Fig. 1).

#### Scans

All participants will have a baseline PET-CT scan before receiving their first cycle of ZA/placebo and a further CT and PET-CT scan after three cycles of treatment are completed. A final CT scan will take place either after the sixth cycle of treatment or at six months from enrolment for those who do not complete the full course of treatment.

### Semi-structured interviews

As a feasibility study paving the way to a larger phase III trial, we are keen to explore patient decisions behind their chosen treatment option. Therefore, we would like to interview a purposive maximum variation sample of ten patients or up to data saturation, which will include participants who are randomised, non-randomised and those who decline participation in the trial altogether. For participants who consent to the trial, this interview will occur at the end of the trial. Those declining participation in the trial will be asked at the time whether they would like to participate in the interview.

### Statistical analysis

The analysis will be according to our feasibility objectives detailed above. The information obtained from this study will allow us to calculate numbers needed to treat in the full trial. Assuming a 40% response rate for chemotherapy alone, the difference in the number of patients with a disease response between the IMP group and the placebo group will be used to calculate the sample size for the full trial. No formal interim analysis is planned; the primary analysis will take place when follow-up is complete for all recruited patients and the database has been locked.

### Safety reporting

Standard definitions and clinical judgement will be used when reporting any adverse events (AE) relating to the trial. Given the nature of the disease and the chemotherapy treatment patients are receiving, certain adverse reactions are to be expected. The expected AEs relating to the IMP and chemotherapy are as listed below. All expected AE and serious adverse event (SAE) data will be captured and reported as appropriate.

Expected AEs relating to ZA:

- Flu-like symptoms
- Nausea
- Poor appetite
- Tiredness
- Sore eyes
- Redness and soreness around drip site
- Electrolyte disturbances (hypocalcaemia/hypomagnesaemia/hypophosphataemia)

Expected AEs relating to chemotherapy:

- Flu-like symptoms
- Tiredness/lethargy
- Nausea and vomiting
- Gastrointestinal upset (diarrhoea)
- Skin reaction
- Peripheral neuropathy
- Pancytopaenia
- Neutropaenic sepsis
- Low folate levels

Expected SAEs relating to ZA and chemotherapy:

- Electrolyte disturbances requiring hospital admission for replacement of electrolytes
- Neutropaenic sepsis requiring hospital admission

### Data collection

All patients approached about the trial and given a PIL will be captured on a screening log. Consent, baseline information and blood results will be recorded on the specific worksheets and subsequently entered onto an electronic database, locally at the relevant sites. At each pre-chemotherapy visit, the patients will have an assessment covering any AEs/SAEs secondary to treatment and have repeat blood tests performed. The results of these and a quality-of-life measure will be documented in the specific worksheets and subsequently entered on to the database. Any CT and PET-CT scans will be pseudo-anonymised to trial number and imported to the local centre for assessment.

The trial team involved with conducting the trial at the lead centre and the statistician will have access to the final trial dataset once the database has been locked down.

### Trial management

A trial steering committee (TSC) comprising the key members of the trial and a patient representative will meet at the beginning, at six months and as necessary thereafter until the trial has closed. An independent data monitoring committee will meet at the start of the trial and six months thereafter to review all safety data and to advice the TSC whether to continue recruiting to the trial.

## Discussion

The Zol-A trial is studying a cohort of patients with an incurable cancer who have limited treatment options. The standard treatment with chemotherapy only has a small effect on prolongation of life hence the need for identifying new treatment options. The objective of this feasibility trial is to gather data from a small number of patients with a diagnosis of MPM and an even smaller number of patients who would be eligible for first-line treatment options. Several MPM treatment trials have terminated early due to poor recruitment (NCT00597116, NCT00003508). It is becoming apparent that even those who would be eligible for chemotherapy are declining the standard treatment due to the poor effectiveness and the toxic side effects associated with the treatment [14]. The semi-structured interviews aim to capture all patient groups involved in the trial to identify reasons behind why patients opt in and out of research trials and chemotherapy treatment. Furthermore, we are looking at their experiences of receiving ZA alongside standard treatment of chemotherapy and the acceptance of this combination.

To obtain efficacy data for the ZA/chemotherapy combination, the number needed to treat is likely to be significantly large. Therefore, considering the outcome of a number of prematurely terminated trials and the large numbers required to obtain any efficacy data with ZA, we embarked on the feasibility trial first. This trial will provide us with radiological information on tumour response after treatment and progression-free survival data which will be used to inform the power calculation when designing the phase III trial. With a disease such as MPM where survival is poor, it is essential to quantify drop-out rates and data completeness rates before designing a full trial and this feasibility trial will assist with estimating these numbers.

The trial design is not without fault. The number of 50 randomised patients in 12 months across three sites is ambitious. The trial will recruit for 13 months in total but for the purpose of our primary feasibility outcome we will look at a 12 consecutive-month period (Additional file 2).

## Trial status

The trial opened to recruitment simultaneously across the three sites in October 2016 and will close to recruitment in November 2017. The study will close when all recruited patients have completed their six-month follow-up appointment.

### Protocol version and date

The protocol published here is version 5.0 dated 19 September 2017. Table 2 contains a list of amendments to date.

**Table 2:** Amendments to date

List of amendments	Summary of change
SA01 14/09/2016	• Personnel randomising to the trial has changed from pharmacy to research members as the randomisation software allows randomisation while protecting the blind • Minor change to inclusion criteria – removed ‘modified RECIST’ from measurable disease section
SA02 25/01/2017	• Change to eligibility criteria – removed ‘measurable disease on CT (tumour thickness > 5 mm)’ • Dr. Steve Walker added as sub-investigator • Radiological data collection is further explained in section 5.13 • Plan of analysis (Section 6.1) details how the radiological information will be used to calculate the sample size for the full study
SA03 26/04/2017	• Addition of Patient appointment schedule v1.0 22/03/17
SA04 08/09/2017	• Request to interview patients who decline participation in the trial • Clarify number of patients for semi-structured interview (up to data saturation rather than the previously stated 10)
NSA01 20/09/2017	• Extend recruitment period by 1 month, to November 2017

## Additional files

Additional file 1: Study visit schedule. (DOCX 160 kb)

Additional file 2: SPIRIT 2013 Checklist: Recommended items to address in a clinical trial protocol and related documents*. (DOC 122 kb)

## Abbreviations

AE: Adverse event
CRP: C-reactive protein
CT: Computed tomography
eGFR: Estimated glomerular filtration rate
IMP: Investigational medicinal product
MDT: Multidisciplinary team
MPM: Malignant pleural mesothelioma
NBT: North Bristol NHS Trust
NHS: National Health Service
PET: Positron emission tomography
PI: Principal investigator
PIL: Patient information leaflet
PS: Performance status
QA: Qualitative analysis
RCT: Randomised controlled trial
SAE: Serious adverse event
TSC: Trial steering committee
UK: United Kingdom
ZA: Zoledronic acid
