# Supplementary figures and images for: Zoledronic acid in the management of mesothelioma - a feasibility study (Zol-A Trial): study protocol for a randomised controlled trial
Source: Trials. 2018 Aug 29;19:467. doi: 10.1186/s13063-018-2851-9 (PMC6116562; doi:10.1186/s13063-018-2851-9)

**
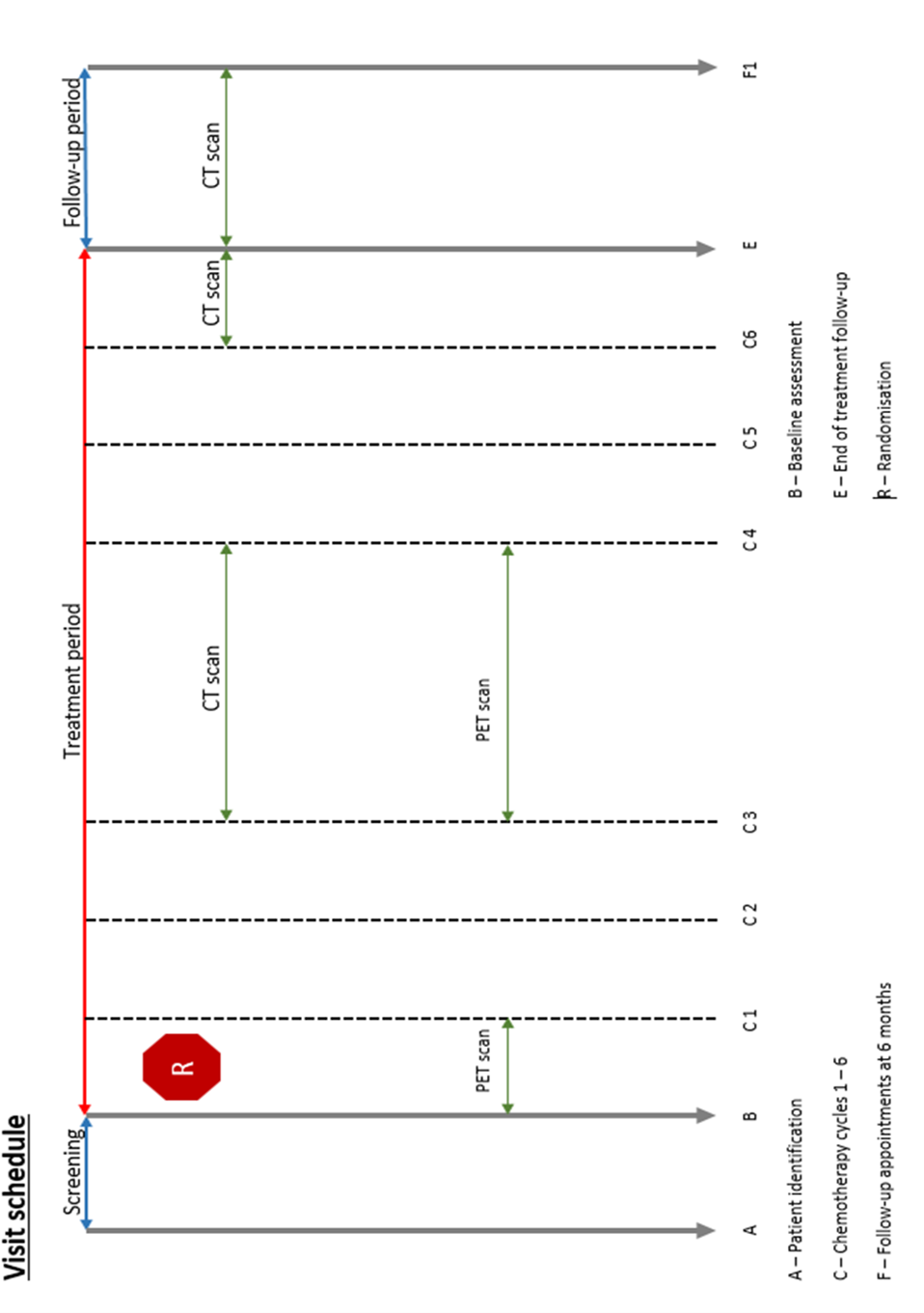
**

Supplement: Supplementary file 1 — Study visit schedule. (DOCX 160 kb) [file 13063_2018_2851_MOESM1_ESM.docx]
